# Comparison of Watermelon and Carbohydrate Beverage on Exercise-Induced Alterations in Systemic Inflammation, Immune Dysfunction, and Plasma Antioxidant Capacity

**DOI:** 10.3390/nu8080518

**Published:** 2016-08-22

**Authors:** R. Andrew Shanely, David C. Nieman, Penelope Perkins-Veazie, Dru A. Henson, Mary P. Meaney, Amy M. Knab, Lynn Cialdell-Kam

**Affiliations:** 1 Human Performance Laboratory, Appalachian State University, North Carolina Research Campus, Kannapolis, NC 28081, USA; niemandc@appstate.edu (D.C.N.); meaneymp@appstate.edu (M.P.M.); 2 Department of Health and Exercise Science, Appalachian State University, Boone, NC 28608, USA; 3 Plants for Human Health Institute, North Carolina State University, Department of Horticulture Science, North Carolina Research Campus, 600 Laureate Way, Kannapolis, NC 28081, USA; penelope_perkins@ncsu.edu; 4 Department of Biology, Appalachian State University, Boone, NC 28608, USA; hensonda@appstate.edu; 5 Kinesiology Department, Queens University of Charlotte, Charlotte, NC 28274, USA; knaba@queens.edu; 6 Department of Nutrition, Case Western Reserve University, Cleveland, OH 44106, USA; lak99@case.edu

**Keywords:** endurance exercise performance, l-citrulline, l-arginine, total nitrate, ferric reducing ability of plasma (FRAP), oxygen radical absorbance capacity (ORAC)

## Abstract

Consuming carbohydrate- and antioxidant-rich fruits during exercise as a means of supporting and enhancing both performance and health is of interest to endurance athletes. Watermelon (WM) contains carbohydrate, lycopene, l-citrulline, and l-arginine. WM may support exercise performance, augment antioxidant capacity, and act as a countermeasure to exercise-induced inflammation and innate immune changes. Trained cyclists (*n* = 20, 48 ± 2 years) participated in a randomized, placebo controlled, crossover study. Subjects completed two 75 km cycling time trials after either 2 weeks ingestion of 980 mL/day WM puree or no treatment. Subjects drank either WM puree containing 0.2 gm/kg carbohydrate or a 6% carbohydrate beverage every 15 min during the time trials. Blood samples were taken pre-study and pre-, post-, 1 h post-exercise. WM ingestion versus no treatment for 2-weeks increased plasma l-citrulline and l-arginine concentrations (*p* < 0.0125). Exercise performance did not differ between WM puree or carbohydrate beverage trials (*p* > 0.05), however, the rating of perceived exertion was greater during the WM trial (*p* > 0.05). WM puree versus carbohydrate beverage resulted in a similar pattern of increase in blood glucose, and greater increases in post-exercise plasma antioxidant capacity, l-citrulline, l-arginine, and total nitrate (all *p* < 0.05), but without differences in systemic markers of inflammation or innate immune function. Daily WM puree consumption fully supported the energy demands of exercise, and increased post-exercise blood levels of WM nutritional components (l-citrulline and l-arginine), antioxidant capacity, and total nitrate, but without an influence on post-exercise inflammation and changes in innate immune function.

## 1. Introduction

The importance of ingesting carbohydrate to maintain blood glucose levels during prolonged, vigorous exercise was recognized at the Boston Marathon in the early 1920s [1,2]. Carbohydrate intake improves endurance performance 2%–6%, lowers perceived exertion, and attenuates post-exercise inflammation 25%–40% [3,4,5,6]. The consumption of multiple transportable carbohydrates (e.g., a mixture of glucose and fructose) during exercise improves the rate of carbohydrate oxidation [7], due to absorption of the carbohydrates by multiple transporters including the sodium-glucose transporter 1 (SGLT1), the universal glucose and fructose transporter (GLUT2), and the fructose transporter (GLUT5) [8]. Data support the use of solutions with a fructose:glucose ratio of 0.8:1 and a consumption rate up to 1.7 g/min [9] to support performance.

Consumption of fruit and fruit juice during endurance exercise as a means of sustaining performance and health is of interest to those desiring natural sources of exogenous carbohydrate. Use of raisins (fructose:glucose ratio of 1.1:1) as the carbohydrate source before and during exercise produced greater rates of carbohydrate oxidation and performance compared to water only [10]. Consuming bananas (fructose:glucose ratio of 1:1) maintained blood glucose during endurance exercise and significantly increased time to exhaustion compared to placebo [11]. Recently, we demonstrated that consuming bananas during a simulated mountainous 75 km cycling time trial resulted in equal performance, maintenance of blood glucose levels, elevated antioxidant capacity, and similar post-exercise inflammation compared to a standard 6% carbohydrate sports drink [12]. These findings were confirmed and extended in a comparison of banana or pear (fructose:glucose ratio of 1:0.44) consumption versus water only during a cycling time trial [13]. Pear consumption during exercise supported performance nearly as well as banana consumption, and both carbohydrate sources resulted in higher blood glucose and carbohydrate oxidation rates, elevated antioxidant capacity, and attenuated post-exercise inflammation compared to water [13]. These data indicate that fruit consumption supports the carbohydrate requirements of prolonged vigorous endurance exercise with the added advantage of augmenting antioxidant capacity.

Watermelon (*Citrullus lanatus*) is a member of the Cucurbitaceae family of gourds and is related to the cucumber, squash, and pumpkin. Watermelon flesh (WM) is ~91% water by weight, and is a rich source of bioavailable compounds including lycopene and other carotenoids, vitamins A and C, and the non-essential amino acid l-citrulline, and is about 6% sugar by weight (fructose:glucose ratio of 1:0.55) [14,15].

Carotenoids are natural fat-soluble compounds that exert antioxidant, anti-inflammatory, and anti-carcinogenic effects [16,17]. Lycopene is the pigment principally responsible for the characteristic deep-red color of watermelon (4532 µg/100 g), and is a highly efficient singlet oxygen quencher [18]. American adults consume 4.5–6.5 mg/day lycopene, of which approximately one-fourth is absorbed in the small intestine, achieving a maximal plasma concentration after about two days with a half-life of nine days [19]. Limited human evidence suggest that lycopene-rich tomato extracts may counter inflammation and oxidative stress following short-term, intensive exercise [20,21].

The amino acid l-citrulline is an endogenous precursor of l-arginine, and nearly all dietary l-citrulline is converted into l-arginine in animals [22]. Nitric oxide (NO) is synthesized from l-arginine by tetrahydrobiopterin (BH4)-dependent NO synthase [22] and l-citrulline supplementation increases NO synthesis [23]. NO increases glucose transporter type 4 (GLUT4 ) translocation and thus glucose flux, which may enhance performance [24]. Acute WM consumption significantly increases l-citrulline and l-arginine plasma levels [25] and chronic WM consumption significantly increases fasting l-arginine plasma levels, but not l-citrulline [26]. The degree to which WM supplementation increases NO levels before and after exercise is currently unknown. Increased fruit ingestion has been linked in several studies to increased antioxidant capacity [27]. Watermelon contains the antioxidants l-citrulline, lycopene, β-carotene, and vitamin C [18,28,29,30,31], and has the potential to increase plasma antioxidant capacity and decrease oxidative stress before and after exercise. To date, two studies using short duration, high intensity exercise protocols have examined the possible ergogenic effects of WM. Acute WM consumption providing ~1.2 g of l-citrulline attenuated moderate muscle soreness in untrained healthy subjects participating in high-intensity exercise intervals, but did not improve performance [32]. Similarly, time to exhaustion during a graded exercise test was not improved following acute consumption of watermelon juice containing ~1 g l-cirulline [33].

Long duration, high-intensity exercise induces significant physiological stress. Given the unique nutritional components in watermelon, we tested the efficacy of WM supplementation before (2 weeks) and during a 75 km cycling time-trial on performance and antioxidant capacity, and as a countermeasure to exercise-induced inflammation and innate immune changes compared to a standard 6% carbohydrate sports beverage.

## 2. Materials and Methods

### 2.1. Subjects

Twenty male cyclists with competitive road racing and time trial experience were recruited from local racing teams. Subjects agreed to train normally, remain weight-stable, and avoid the use of large-dose vitamin/mineral supplements (above 100% of recommended dietary allowances), herbs, and medications known to affect inflammation and immune function during the study. Informed consent was obtained from each subject and all study procedures were reviewed and approved by the Appalachian State University Institutional Review Board.

### 2.2. Study Design and Procedures

Two weeks prior to the first 75 km time trial, each subject completed study orientation and baseline testing in the North Carolina Research Campus Human Performance Laboratory operated by Appalachian State University. During study orientation subjects provided demographic information and training histories and were instructed to follow a diet moderate in carbohydrate (using a provided food list) during the 3 day period before each 75 km time trial.

During baseline testing, maximal cardiorespiratory fitness and body composition were measured. A cycle ergometry protocol (beginning at 150 W, 25 W increase per 2 min stage) was used to measure maximal power on a Lode cycle ergometer (Lode Excaliber Sport, Lode B.V., Groningen, The Netherlands) and peak oxygen consumption (VO_2peak_) with a Cosmed Quark CPET metabolic cart (Rome, Italy) [12]. Heart rate was measured using a Polar Heart Rate Monitor (Polar Electro Inc., Woodbury, NY, USA). Body composition was measured with the BodPod system (Life Measurement, Concord, CA, USA). The environmental conditions were maintained at 19–20 °C and 45%–55% relative humidity; each subject had a fan directed on them to ensure consistent air flow during the time trial.

Subjects were randomized to either the watermelon (WM) or the 6% carbohydrate beverage (CHO) condition for the first 75 km time trial and then crossed over to the opposite condition for the second time trial with a 2-week washout period between trials. Subjects randomized to the WM trial were provided a 2-week supply of frozen WM puree. The subjects maintained the containers of the frozen WM in a standard freezer (−20 °C) and rapidly thawed each container of WM puree under hot running water prior to drinking it. Slowly thawing non-pasteurized WM results in a poor taste. However, thawing it quickly under hot water results in a more normal taste. The amount lycopene and l-citrulline do not change appreciably with a single freeze/thaw cycle. The subjects consumed 980 mL WM puree per day (equivalent of 60.7 g total sugar, 1.47 g l-citrulline, 0.465 g l-arginine, 44.4 mg lycopene, 5576 IU vitamin A, 0.44 IU vitamin B-6, and 79.4 IU vitamin C [15,26,34]) during the 2 weeks period prior to the WM trial. Watermelon puree for the study was prepared at Blue Ridge Venture Foods (Candler, NC, USA) from the seedless variety Crunchy Red harvested in eastern North Carolina. Watermelon flesh was pureed using a screw finisher with a 0.1 mm diameter stainless steel screen tube. The puree was bottled in 1 L capacity polyethylene containers and flash frozen without filtration or pasteurization. The frozen WM puree was maintained at −20 °C.

The morning of the 75 km time trial, subjects consumed the WM puree (980 mL) (or no WM puree) and then a standardized meal at 12:00 p.m. using Boost Plus at 10 kcal/kg (41.9 kJ/kg) (Boost Plus; Mead Johnson Nutritionals, Evansville, IN, USA). Subjects reported to the lab at 2:45 p.m. and then provided a blood sample. At 3:20 p.m., subjects ingested 0.4 g/kg carbohydrate from WM or from a standard 6% CHO beverage (Gatorade™, Chicago, IL, USA). Subjects ingested 0.2 g/kg of carbohydrates body weight every 15 min of WM or 6% CHO beverage during the 75 km time trials.

Subjects cycled (3:30 p.m. start) the mountainous 75 km time trial course [12] on their own bicycles on CompuTrainer Pro Model 8001 trainers (RacerMate, Seattle, WA, USA). Workload was continuously monitored using the CompuTrainer MultiRider software system (version 3.0, RacerMate, Seattle, WA, USA). Heart rate and rating of perceived exertion (RPE) were recorded every 30 min. Pre- and post-exercise fingertip capillary blood samples were drawn and analyzed using the YSI 2300 STAT Plus Glucose and Lactate analyzer (Yellow Springs, OH, USA). Blood samples were taken via venipuncture post-exercise and 1 h post-exercise. Subjects answered questions on digestive health using a 12-point Likert scale (1 relating to “none at all”, 6 “moderate”, and 12 “very high”).

### 2.3. Analytical Measures

#### 2.3.1. Complete Blood Count

Routine complete blood counts with white blood cell differential counts were made (Coulter Ac. T™ 5Diff Hematology Analyzer, Beckman Coulter, Inc., Miami, FL, USA) for the determination of plasma volume change and leukocyte subtypes for the immune function assay [35].

#### 2.3.2. Plasma Cytokines

The total plasma concentration of six inflammatory cytokines (tumor necrosis factor α (TNFα), interleukins 6, 8, and 10 (IL-6, IL-8, IL-10), monocyte chemoattractant protein-1 (MCP-1), and granulocyte colony-stimulating factor (G-CSF)) was determined using an electrochemiluminescence based solid-phase sandwich immunoassay (Meso Scale Discovery, Gaithersburg, MD, USA) [12,36]. All samples and provided standards were analyzed in duplicate; the intra-assay CV ranged from 1.7% to 7.5% and the inter-assay CV ranged 2.4% to 9.6% for the cytokines measured. The minimum detectable concentration of IL-6 was 0.27 pg/mL, TNFα 0.50 pg/mL, GM-CSF 0.20 pg/mL, IFNγ 0.53 pg/mL, IL-1β 0.36 pg/mL, IL-2 0.35 pg/mL, IL-8 0.09 pg/mL, and IL-10 0.21 pg/mL. Pre- and post-exercise samples for the cytokines were analyzed on the same assay plate to decrease inter-kit assay variability.

#### 2.3.3. Granulocyte and Monocyte Phagocytosis, Oxidative Burst Activity

Granulocyte and monocyte phagocytosis (GR-PHAG, MO-PHAG), oxidative burst activity (GR-OBA, MO-OBA) were assayed as previously described by Meaney et al. [37]. Briefly, phagocytosis was measured through the uptake of fluorescein isothiocyanate (FITC)-labeled *Staphylococcus aureus* bacteria and oxidative burst was measured through the oxidation of nonfluorescent hydroethidine (HE) to fluorescent ethidium bromide in cells stimulated with unlabeled bacteria. Samples were processed on a Q-Prep™ Workstation (Beckman Coulter, Inc.) and analysis was performed within 18 h of blood collection using a Beckman Coulter FC10 500 flow cytometer. After gating on the granulocyte and monocyte populations using forward scatter and side scatter, the mean fluorescence intensity (MFI; x-mean) and percent positive cells for FITC (FL1) and oxidized HE (FL2) were determined.

#### 2.3.4. Plasma Antioxidant Capacity

Plasma antioxidant capacity was determined by two independent measures; the ferric reducing ability of plasma (FRAP) assay and the oxygen radical absorbance capacity (ORAC). The FRAP assay, a single electron transfer reaction, was conducted as previously described [30,36,38]. The FRAP assay utilizes water-soluble antioxidants native to the plasma collected from EDTA-treated blood to reduce ferric iron to the ferrous form subsequently producing a chromogen identifiable at 593 nm (Synergy H1 Hybrid Reader, BioTek Instruments Inc., Winooski, VT, USA). Samples and standards are expressed as ascorbate equivalents based on an ascorbate standard curve. Intra-assay and inter-assay coefficients of variation (CVs) were less than 5% and 7%, respectively. Plasma antioxidant power was also measured by the ORAC assay using methods previously described [36]. The ORAC assay depends on exogenous peroxyl radicals generated by 2,2′-azobis (2-methylpropionamide) dihydrochloride (AAPH) to oxidize fluorescein. Antioxidants in blood plasma delay oxidation of the fluorescent probe. Samples and standards are expressed as Trolox equivalents (μmol/L) based on a Trolox standard curve calculated by a fluorescence plate reader (Synergy H1 Hybrid Reader) as area under the curve. Intra and inter-assay CVs for ORAC were 4% and 7%, respectively.

#### 2.3.5. Plasma Amino Acid Analysis

The amino acid concentration of plasma collected from heparin-treated blood was determined according to the methods of Wu and Meininger [39]. Briefly, 50 μL of plasma was mixed with 50 μL of 1.5 M HClO_4_. To this, 1.125 mL of HPLC-grade water and 25 μL of 2 M K_2_CO_3_ were added. The mixture was centrifuged (10,000× *g* for 1 min) and the supernatant was analyzed by HPLC using a Supelco C18 column (Supelco, Bellefonte, PA, USA) and a Waters HPLC system (Waters, Milford, MA, USA). Amino acids in samples were quantified on the basis of standards (Sigma Chemicals, St. Louis, MO, USA).

#### 2.3.6. Total Nitrate

The total nitrate concentration of plasma collected from EDTA-treated blood was determined fluorometrically according to the manufacture’s protocol (#780051; Cayman Chemical Company, Ann Arbor, MI, USA). Immediately prior to conducting the assay, the plasma was filtered according to the manufacturer’s recommendation (#UFC801096, Millipore, Billerica, MA, USA). All samples and standards were analyzed in triplicate. The minimum detectable limit of the assay is 30 nM nitrite. Pre- and post-exercise samples were analyzed on the same assay plate to decrease inter-kit assay variability.

### 2.4. Statistical Analysis

All data are expressed as mean ± SEM. The biomarker data were analyzed using a 2 (condition) × 3 (time) repeated-measures ANOVA, within-subject design. When interaction effects were significant (*p* ≤ 0.05), changes between baseline and each pre-exercise condition and pre-exercise, post-exercise, and 1 h post-exercise time points within Watermelon or CHO conditions were compared between trials using 2-tailed paired *t*-tests, with significance set after Bonferroni adjustment at *p* ≤ 0.0125.

## 3. Results

Twenty subjects completed the study; subject characteristics are summarized in Table 1. Mean power (192 ± 9.2, 198 ± 9.1 watts; *p* = 0.203), heart rate (87.2% ± 1.10%, 85.5% ± 1.13% HR_max_; *p* = 0.111), and total time (2.74 ± 0.35, 2.68 ± 0.36 h; *p* = 0.192) did not differ between WM and CHO 75 km time trials, respectively. Subjects reported a slightly higher rating of perceived exertion (16.8 ± 0.29, 16.2 ± 0.23 RPE units; *p* = 0.030) at the conclusion of the WM trial than the CHO trial, respectively. The pattern of increase in blood glucose (30.2%, 29.2%; interaction effect *p* = 0.959) and blood lactate (294%, 314%; interaction effect *p* = 0.248) did not differ between WM and CHO trials. Mean carbohydrate intake during the WM and CHO trials was 182 ± 9.79 grams and did not differ between trials (*p* = 0.178). The volume of WM puree consumed during the trial did not differ from volume of CHO beverage consumed (2.98 ± 0.17 L, 2.86 ± 0.16 L; *p* = 0.098). Compared to the CHO condition, subjects reported feeling fuller (*p* = 0.0148) but not more bloated (*p* = 0.226) after consuming WM during the time trial. Subjects lost more body mass during the WM condition (−0.60 ± 0.58 kg, −0.01 ± 0.60 kg; *p* = 0.002) than during the CHO condition, respectively, but the pre- to post-exercise change in plasma volume did not differ (6.49% ± 4.08%, 6.30% ± 2.25%; *p* = 0.970).

The acute inflammatory response to completing the 75 km time trial did not differ appreciably between WM and CHO trials (Table 2). The pattern of increase in the plasma cytokine G-CSF was greater after the WM trial than the CHO trial (Table 2); however, post hoc analysis did not reveal differences (*p* > 0.0125). Exercise-induced changes in innate immune function did not differ between WM and CHO trials (Table 2). The pattern of increase in GR-PHAG and MO-PHAG and GR-OBA and MO-OBA did not differ between WM and CHO (Table 2).

**Figure 1:**
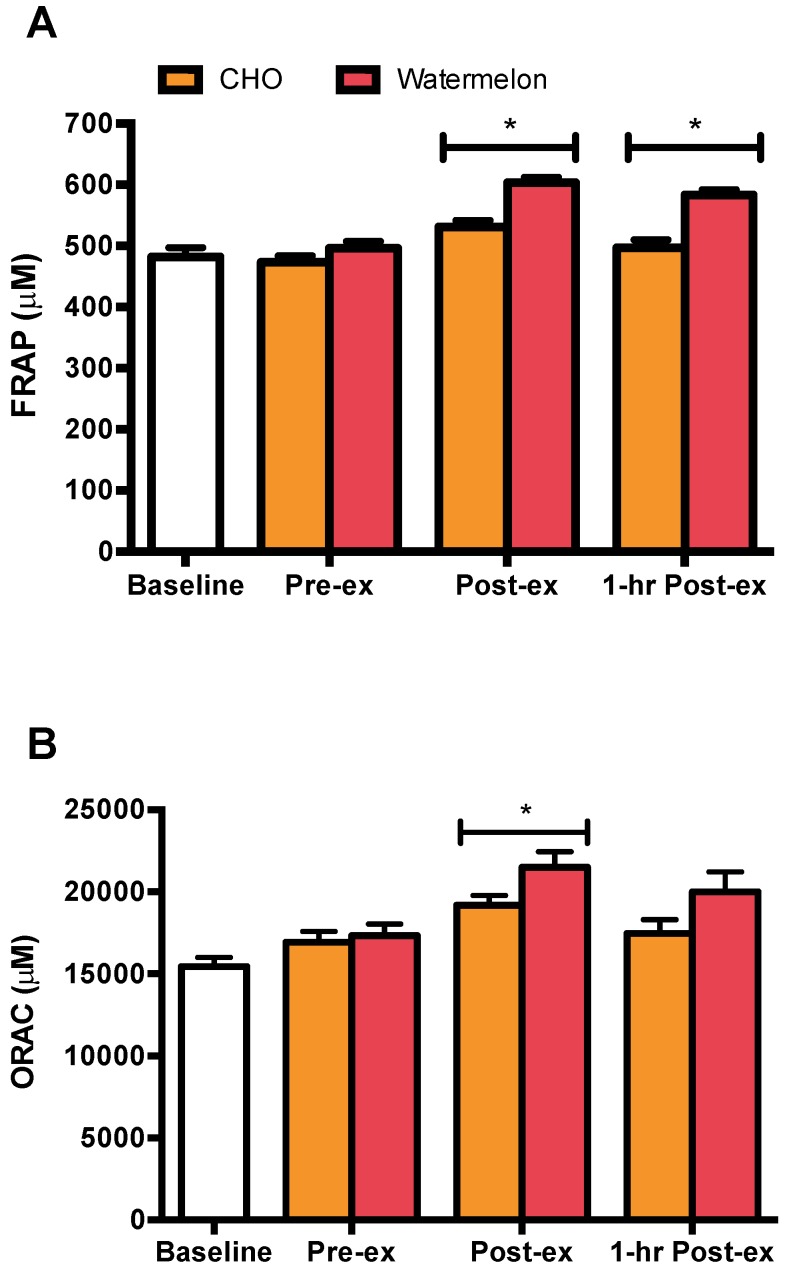
Watermelon consumption during exercise potentiates the exercise-induced increase in plasma antioxidant capacity. (**A**) Plasma FRAP = ferric reducing ability of plasma (expressed as ascorbate equivalents) and (**B**) plasma ORAC = oxygen radical absorbance capacity (expressed as trolox equivalents) were higher in WM compared to CHO following 75 km cycling (interaction effect, *p* < 0.001, each); * *p* < 0.0125 compared to time matched CHO.

The exercise-induced patterns of change in plasma antioxidant capacity were greater in WM compared to CHO trials. In contrast to the CHO trial, WM consumption resulted in a significantly greater pre- to post-exercise and pre- to 1 h post-exercise increase in plasma FRAP (interaction effect *p* < 0.001) (Figure 1A). Similarly, the pre- to post-exercise pattern of increase in plasma ORAC was greater in WM than CHO (interaction effect *p* < 0.001) (Figure 1B).

**Figure 2:**
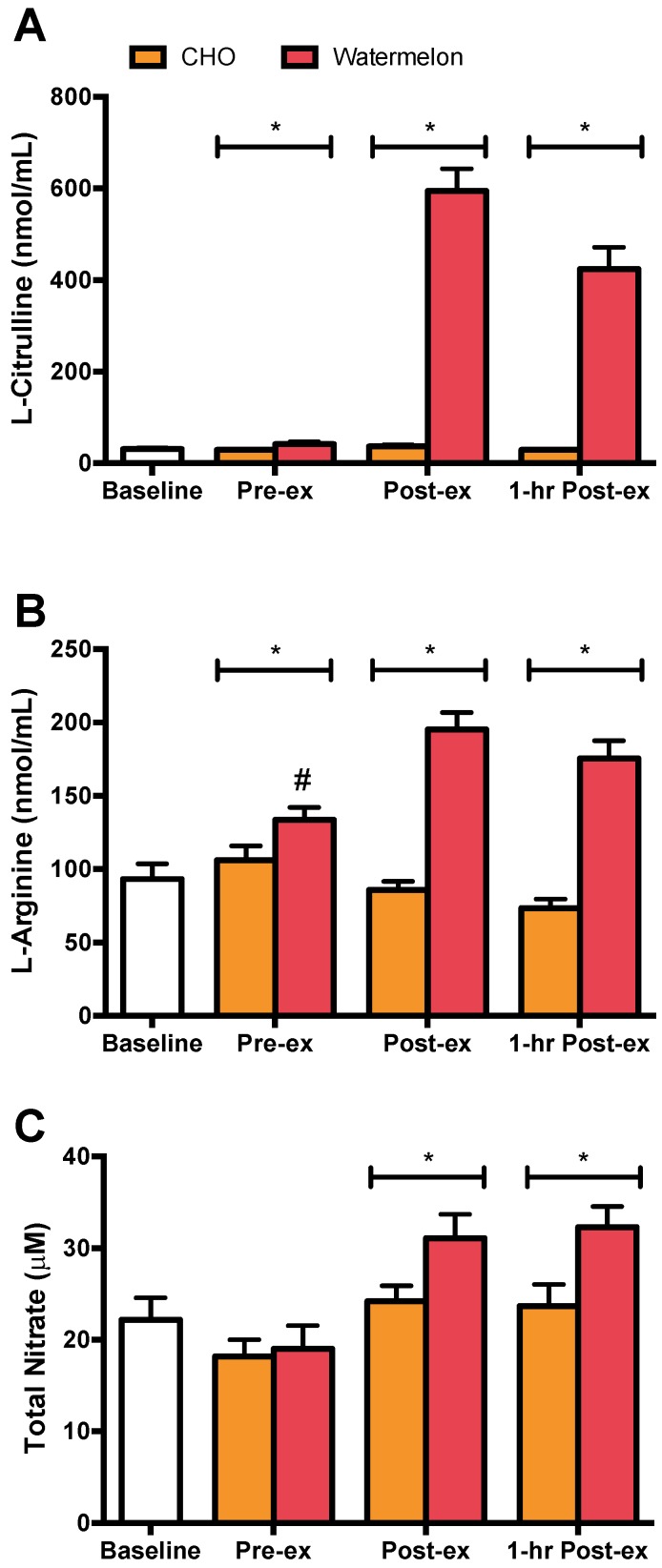
Watermelon consumption increases plasma l-citrulline (interaction effect *p* < 0.001), l-arginine (interaction effect *p* < 0.001), and total nitrate (interaction effect *p* = 0.004). Plasma concentrations of (**A**) l-citrulline; (**B**) arginine; and (**C**) total nitrate; # *p* < 0.0125 compared to baseline; * *p* < 0.0125 compared to time matched CHO.

The pattern of increase in plasma l-citrulline differed between WM and CHO trials (interaction effect *p* < 0.001) with differences measured between conditions after 2 weeks WM ingestion, immediately post-, and 1 h post-exercise (Figure 2A). The pattern of increase in plasma l-arginine differed between WM and CHO trials (interaction effect *p* < 0.001) with an increase from baseline, and differences measured between trials after 2 weeks WM ingestion, immediately post-, and 1 h post-exercise (Figure 2B). The pattern of increase in plasma total nitrate differed between WM and CHO trials (interaction effect *p* = 0.004) with differences measured between conditions immediately post-, and 1 h post-exercise (Figure 2C).

## 4. Discussion

This randomized, crossover study investigated the effect of WM puree consumption for two weeks before and during a bout of vigorous exercise (relative to matched carbohydrate beverage ingestion) on exercise performance and antioxidant capacity, and as a countermeasure to exercise-induced inflammation and innate immune changes in trained cyclists. The 75 km cycling time trial was associated with the typical increases in plasma cytokines and granulocyte and monocyte phagocytosis (a marker of post-exercise inflammation) measured during previous trials with carbohydrate-fed athletes [12,40]. Despite significantly higher plasma antioxidant capacity, l-arginine, nitrate, and citrulline, WM ingestion was not associated with alterations in the pattern of change in inflammation and immune measures. Performance times were comparable between WM and CHO beverage ingestion, supporting previous findings in our lab that high-fructose fruit ingestion supports intensive, long duration exercise to the same degree as sports beverages [12,13].

WM benefits in attenuating post-exercise inflammation may be measurable during extended periods of heavy training and not after one exercise challenge event. The water content (91%) and unique combination of nutritional components found in WM are of interest to athletes seeking a natural whole food source for hydration and nutrition during physical activity. Lycopene is the major carotenoid (84%–97%) in red WM flesh, and one serving (280 g) contains 14–22 mg of lycopene [41]. WM is high in fructose, and each serving contains 3.4 g sucrose, 4.4 g glucose, and 9.4 g of fructose. Watermelon is one of the richest food sources of l-citrulline, a non-essential amino acid, and contains a small amount of l-arginine, an essential amino acid. Each serving of WM (286 g) provides 0.429 g of l-citrulline, 0.135 g of l-arginine, and small amounts of other amino acids [15,26,34]. WM has moderate amounts of potassium and vitamin C. In general, the distinctive mixture of nutritional components in WM led us to hypothesize that 2 weeks ingestion would alter post-exercise cytokine and immune measures, but these effects did not emerge within the context and limitations of this study. We have previously shown that carbohydrate compared to water ingestion during 75 km cycling trials results in muted post-exercise inflammation, and this study would have been strengthened had the research design included a water-only condition. Nonetheless, contrary to our hypothesis, WM ingestion did not add to the well-known anti-inflammatory effects associated with carbohydrate ingestion during exercise [13,40].

To our knowledge this is the first study to determine the effectiveness of WM to support the energy demands of vigorous endurance (>2 h) exercise. The amount and timing of the carbohydrate provided to our subjects was based on the American College of Sports Medicine recommendation, 30–60 g/h or 0.7 g/kg/h delivered every 15–20 min [42]. The fructose:glucose ratio of WM is 1:0.55 [14]. Although gut absorption [43], and thus the rate of oxidation [44], of exogenous fructose is lower than glucose, the blood glucose and lactate data and performance measures (time and average power output) indicate that the dose of WM utilized in this study prevented exercise-induced hypoglycemia and supported the energy demands of vigorous endurance cycling. The current data support our previous findings wherein providing exogenous carbohydrate via bananas and pears maintained blood glucose levels and supported the energy demands of vigorous cycling [12,13]. Tarazona-Diaz et al. [32] found that acute WM supplementation did not enhance anaerobic cycle ergometer work capacity. Cutrufello et al. [33] recently reported that a single acute dose of WM did not enhance strength, anaerobic threshold, time to exhaustion or VO_2max_. These two WM studies utilized short-duration, high-intensity exercise bouts that are not limited by blood glucose or glycogen levels.

The rate and total volume of beverage consumed did not differ between trials. However, the subjects reported feeling significantly fuller during the WM trial. In our previous studies, we reported that subjects consuming bananas and pears felt fuller and more bloated, but without influencing RPE [12,13]. The fiber content of WM and fruit more than likely contributed to the perception of feeling fuller, and may have contributed to the small but significantly higher RPE during the WM trial. The subjects lost approximately 0.5 kg (0.62%) more body mass during the WM trial than the CHO trail; similar to what we previously reported when bananas were consumed during prolonged exercise [12]. The loss in body mass did not result in a greater pre- to post-exercise change in plasma volume between trials. For most individuals, a loss of >2% body mass may hinder aerobic performance due to dehydration [45]. Consuming WM puree, within the conditions employed in this study, met the hydration needs of the cyclists.

WM ingestion was associated with increases in both measures of plasma antioxidant capacity (FRAP and ORAC). These data support our previous findings with banana and pear [12,13]. The increase in plasma antioxidant capacity, especially post-exercise, may be attributed to an increase in the plasma concentration of uric acid [30,46]. During fatiguing exercise, active skeletal muscle oxidation of purines increases, thus increasing the efflux of uric acid from skeletal muscle into the blood compartment [47]. However, hydrogen peroxide is generated at two steps of this biochemical process, consequently, the exercise-induced increase in plasma uric acid may not be a mechanism of compensatory antioxidant enhancement as some speculate. Independent of the exercise-induced increase in plasma uric acid, the greater amount of fructose consumed during the WM trial may have resulted in a larger increase in hepatic uric acid production. In the liver, metabolism of fructose to fructose 1-phosphate through fructokinase results in production of uric acid [48]. While an acute decrease in uric acid does not exacerbate exercise-induced oxidative stress in the blood or hinder exercise performance [49], the benefit of WM-derived uric acid did not improve performance in this study. Consumption of l-citrulline, a hydroxyl scavenger, and other antioxidants present in WM (lycopene, β-carotene, and vitamin C), may have further contributed to the increased total antioxidant capacity of the plasma [18,28,29,30,31]. The performance benefit of acutely increasing the total antioxidant capacity of plasma is unclear [50] and remains an interesting area of study.

With the exception of G-CSF, the pattern of change in the markers of systemic inflammation and immune function differed little between the WM and CHO. The WM trial post-exercise and 1 h post-exercise increase in G-CSF was 15% and 18% greater, respectively. In response to intense exercise G-CSF mediates mobilization of progenitor cells [51] and prevents neutrophil apoptosis and stimulates neutrophil release [52,53]. The mechanism by which WM consumption potentiated the G-CSF response to exercise warrants further study. Watermelon provides a significant amount of l-citrulline and a previous report suggests that citrulline-malate modulates polymorphonuclear neutrophil function. Sureda et al. [23] supplemented competitive cyclists with 6 g of citrulline-malate prior to a competitive 3 h cycling race. Post-exercise polymorphonuclear neutrophils from subjects in the supplemented condition had significantly greater reactive oxygen species levels than baseline, indicating that acute citrulline-malate may attenuate exercise-induced immune dysfunction through an NO-dependent mechanism [23]. In the current study total nitrate, a proxy measure of NO production, significantly increased during the WM trial in similar fashion to the report by Sureda et al. [23], but without an effect on oxidative burst activity.

l-citrulline and l-arginine have been studied as potential ergogenic aids. Nitric oxide (NO) synthase, in conjunction with specific cofactors, converts l-arginine into NO and l-citrulline. Further, l-citrulline may be converted to l-arginine via argininosuccinate synthase [22]. Putatively, enhancing NO metabolism would improve performance through greater blood flow and increasing glucose uptake via enhance GLUT4 translocation in working skeletal muscle [24]. During the 2-week intervention period subjects consumed 980 mL of WM/ day, the equivalent of 1.47 g l-citrulline and 0.465 g l-arginine/day and approximately 4.39 g l-citrulline and 1.41 g l-arginine during the 75 km time trial. Plasma l-citrulline concentrations return to baseline values approximately 3–5 h after ingestion [54] and the small but non-significant elevation in pre-exercise plasma concentration is attributable to the 980 mL dose of WM consumed the morning of the WM trial. Following consumption of l-citrulline, plasma l-arginine concentrations take approximately 8 h to return to baseline [54]. The significant pre-exercise increase in plasma l-arginine is the result of consuming the WM during the previous supplementation period [26] and the morning of the WM time trial. Our baseline and pre-exercise plasma l-citrulline and l-arginine data are in agreement with Collins et al. [26] and Mandel et al. [25].

Subjects completed the WM trial in less than 3 h and consumed ~3 L of WM. This resulted in a 14-fold and a 32% increase in the pre- to post-exercise plasma concentration of l-citrulline and l-arginine, respectively. To our knowledge only one study has measured plasma l-citrulline and l-arginine concentrations after consuming one dose of WM [25]. In this study [25] subjects consumed a single 3.3 kg serving of WM. One hour post-WM consumption, plasma l-citrulline and l-arginine concentrations increased 27-fold and 3-fold, respectively, and returned to baseline by the 8-h time point. The smaller increase in plasma l-citrulline and l-arginine in our study was due to the smaller amount of WM consumed and the greater amount of time over which our subjects consumed the WM. Short-duration high-intensity exercise is not improved by a single dose of l-citrulline or l-citrulline supplemented over a 24 h period [33,55]. Recent evidence suggests that l-citrulline supplementation over a 1-week period can increase performance when exercising at a high percentage of the subject’s VO_2max_ for approximately 10 min [56,57]. l-citrulline supplementation is postulated to improve performance by enhancing NO-dependent vasodilation, and thus blood flow, and increased mitochondrial respiration during exercise [56]. The plasma concentration of total nitrate, a proxy measure of NO production, increased significantly more during the WM trial compared to the CHO trial, 63% and 33%, respectively. The lack of improved performance during the WM trial, despite the apparent increase in NO production, suggests that NO-dependent mechanisms did not limit exercise performance in the current study.

## 5. Conclusions

Our data indicate that ingestion of watermelon (WM) puree is as effective as a 6% CHO beverage in supporting endurance exercise performance, with the added advantage of improving antioxidant capacity through increased intake of lycopene, l-citrulline, and vitamins A and C. While the RPE was greater during the WM trial this did not dampen time trial performance. Changes in blood glucose, lactate, inflammation, antioxidant capacity, and innate immune measures were comparable between WM puree and 6% CHO beverage 75 km cycling trials, and similar to what we have previously reported for CHO-fed athletes. WM puree ingestion during exercise increased plasma l-citrulline, l-arginine, and total nitrate, but without discernable acute effects on post-exercise inflammation and innate immune function relative to CHO.

## Figures and Tables

**Table 1:** Subject characteristics (*n* = 20).

Variable	Mean ± SEM
Age (year)	48.5 ± 2.3
Body mass (kg)	81.04 ± 2.2
% Body fat	19.6 ± 1.5
BMI (kg/m^2^)	25.1 ± 0.7
Years cycling	10.95 ± 2.3
Watt_max_	314 ± 9.8
Peak oxygen consumption (VO_2peak_, mL·kg^−1^·min^−1^)	51.5 ± 1.9

Data are means ± SEM; BMI = body mass index; W = watts.

**Table 2:** Inflammation and immune-function markers.

Variable	Baseline	Pre-Exercise	Post-Exercise	1-h Post-Exercise	Time; Interaction *p* Values
Inflammatory markers					
WBC (10^9^/L)	6.2 ± 0.34				
CHO		5.74 ± 0.31	12.2 ± 0.96	10.9 ± 0.83	<0.001; 0.125
WM		5.58 ± 0.39	14.7 ± 1.01	12.4 ± 0.85	
TNF-α (pg/mL)	10.5 ± 1.03				
CHO		10.2 ± 0.92	12.8 ± 1.11	11.9 ± 1.14	0.005; 0.936
WM		10.2 ± 0.99	12.3 ± 1.29	12.1 ± 1.19	
IL-6 (pg/mL)	1.02 ± 0.28				
CHO		0.80 ± 0.13	10.2 ± 1.83	7.92 ± 1.58	<0.001; 0.921
WM		0.71 ± 0.14	9.98 ± 1.63	7.68 ± 1.56	
IL-8 (pg/mL)	3.21 ± 0.33				
CHO		3.26 ± 0.37	10.9 ± 1.01	12.2 ± 1.55	<0.001; 0.506
WM		3.59 ± 0.33	12.1 ± 1.52	11.4 ± 1.55	
IL-10 (pg/mL)	2.31 ± 0.44				
CHO		2.46 ± 0.48	10.5 ± 3.83	8.44 ± 2.82	0.002; 0.292
WM		2.93 ± 0.82	15.5 ± 5.68	16.0 ± 6.64	
MCP-1 (pg/mL)	194 ± 7.80				
CHO		188 ± 8.99	344 ± 23.8	333 ± 27.3	<0.001; 0.206
WM		188 ± 9.44	375 ± 21.1	338 ± 16.9	
G-CSF (pg/mL)	9.47 ± 0.56				
CHO		9.94 ± 0.97	16.2 ± 1.55	16.8 ± 1.53	<0.001; 0.041
WM		10.7 ± 0.96	18.7 ± 2.12	19.9 ± 1.94	
GR-PHAG (MFI)	49.7 ± 4.20				
CHO		32.7 ± 2.81	63.2 ± 8.83	75.3 ± 11.9	0.001; 0.635
WM		40.8 ± 6.75	72.2 ± 12.0	83.6 ± 16.3	
MO-PHAG (MFI)	26.5 ± 1.64				
CHO		20.1 ± 1.50	36.0 ± 3.46	44.4 ± 5.18	<0.001; 0.612
WM		23.9 ± 3.89	39.2 ± 4.87	44.4 ± 6.88	
GR-OBA (MFI)	23.3 ± 1.11					
CHO		16.3 ± 1.35	24.6 ± 2.08	26.3 ± 2.78	<0.001; 0.612
WM		18.5 ± 2.54	26.2 ± 3.28	23.8 ± 3.47	
MO-OBA (MFI)	11.8 ± 0.44				
CHO		9.70 ± 0.59	13.5 ± 0.73	14.7 ± 0.98	<0.001; 0.173
WM		10.6 ± 1.15	13.1 ± 1.09	13.0 ± 1.09	

Data are means ± SEM; WBC = Total blood leukocytes; TNF = Tumor Necrosis Factor; IL = interleukin; MCP = monocyte chemo attractant protein; granulocyte colony-stimulating factor = G - CSF; MFI = mean fluorescence intensity; GR = granulocyte; PHAG = phagocytosis; MO = monocyte; OBA = oxidative burst activity.
